# 小细胞肺癌维持治疗的研究进展

**DOI:** 10.3779/j.issn.1009-3419.2015.09.06

**Published:** 2015-09-20

**Authors:** 云云 陆, 云 范

**Affiliations:** 310053 杭州，浙江省肿瘤医院肿瘤内科 Department of Medical Oncology, Zhejiang Provincial Cancer Hospital, Hangzhou 310053, China

**Keywords:** 肺肿瘤, 维持治疗, 化疗, Lung neoplasms, Maintenance therapy, Chemotherapy

## Abstract

肺癌是世界上最常见的恶性肿瘤之一，其中小细胞肺癌发生率约15%。小细胞肺癌对一线化疗高度敏感，但多数患者在一线治疗缓解后很快出现复发进展，因此，自20世纪80年代起已开展不少小细胞肺癌维持治疗的相关临床研究，涉及到化疗药物、生物制剂及分子靶向药物。但其结果仍存在争论，现对该方面内容综述如下。

## Abstract (en)

Lung cancer is one of the common malignant tumors in the world, the incidence of small cell lung cancer is about 15% among them. Small cell lung cancer is highly sensitive to first-line chemotherapy, but most of the patients relapse after the first-line therapy quickly. Despitemany clinical researchof chemotherapy, biological agents and molecular targeting agents since the 1980s, there still remains a substantial lack of consensus regarding the appropriate therapeutic management on maintenance therapy of small cell lung cancer. The review focuses on maintenance therapy of small cell lung cancer.

肺癌是中国发病率和死亡率最高的恶性肿瘤^[1]^，其中小细胞肺癌（small cell lung cancer, SCLC）约占肺癌总数的15%^[2]^。SCLC的生物学特点是癌细胞分化差、增殖快，具有高度侵袭性，因此临床上容易出现早期扩散转移。SCLC对化放疗高度敏感，但很多患者在一线治疗缓解后很快出现复发进展，且复发后易出现耐药^[3]^。因此维持治疗的理念非常契合SCLC的治疗需求，从20世纪80年代起就开始了SCLC维持治疗的相关临床研究，涉及到化疗药物、生物制剂及分子靶向药物的维持治疗，但至今，有关SCLC的维持治疗尚无明确结论。

## 1 维持治疗概述

维持治疗是指患者在完成一定疗程的标准一线方案治疗后，如果疾病无进展且体力状态评分良好，再接受药物治疗，以期取得更长时间的疾病缓解和生存。维持治疗的药物通常采用一线化疗方案中的一种或几种药物（定义为同药维持），或是与一线化疗药物无交叉耐药的另一种药物或方案（定义为换药维持）。在没有严重毒性反应前提下，维持治疗将一直进行至疾病进展时（晚期患者常采用）或某个设定的时间点。其理论基础来源于Goldie和Coldman^[4]^假设，即尽早使用非交叉抑制药物可以在耐药产生前增加杀伤肿瘤细胞的效能，使治疗效果最优化。目前，依据所用维持药物的不同，可将SCLC的维持治疗研究分为化疗药物维持、免疫治疗维持及分子靶向药物维持。

## 2 化疗药物的维持治疗

### 2.1 原方案维持化疗

由于SCLC对一线化疗高度敏感，早期维持化疗的研究主要集中在6个疗程一线化疗后继续原方案维持，亦即单纯地延长治疗周期，严格地说，这种形式不能称为真正意义上的维持治疗。1986年Cullen等^[5]^率先报道309例SCLC患者接受6个疗程的长春新碱联合阿霉素及环磷酰胺方案诱导治疗。其中，61例治疗有效的广泛期SCLC（extensive disease-SCLC, ED-SCLC）患者随机进入原方案维持组或观察组，结果显示维持治疗组有更长的生存时间（372天*vs* 259天，*P*=0.006）；而32例局限期SCLC（limited disease-SCLC, LD-SCLC）患者进行原方案维持与否的随机对照研究，两组患者的生存时间（overall survival, OS）无明显差异；结论认为ED-SCLC患者如果诱导治疗有效，继续原方案维持治疗可延长生存。但此后发表的有关单纯延长治疗周期的随机对照研究均不支持此结论。如1989年有报道在65例SCLC患者中随机比较6个疗程与12个疗程的化疗（化疗方案:依托泊苷、环磷酰胺、氨甲喋呤和长春新碱），结果表明超过6个疗程的化疗未给患者带来临床获益。有研究^[6]^于1993年报道在687例SCLC患者中随机比较5个疗程与12个疗程的CDE方案（环磷酰胺、阿霉素及依托泊苷），434例患者可评价疗效，12个疗程组的中位无进展生存时间（progression-free survival, PFS）较5个疗程组延长2个月（177天*vs* 114天，*P*=0.000, 4），但两组患者的OS无明显差异，作者认为延长化疗周期并不能带来生存时间的延长。其他相关研究亦得出类似结论。总而言之，单纯延长治疗周期，患者的生存获益不明显，毒副反应却较大，患者的生活质量受影响。因此，这种维持治疗模式逐渐被摒弃，换药维持或单药维持的研究成为主流。

### 2.2 同药或换药维持化疗

依托泊苷或伊立替康联合铂类（顺铂或卡铂）是ED-SCLC的标准一线化疗方案；拓扑替康是唯一有适应证的二线化疗药物。故SCLC维持化疗多数采用上述化疗方案或药物。

1988年Einhorn等^[7]^报道了160例LD-SCLC经VAC方案（环磷酰胺、阿霉素和长春新碱）诱导化疗加胸部放疗后疾病控制的患者随机进入2个疗程依托泊苷联合顺铂方案巩固治疗组或观察组，结果发现巩固治疗组较观察组患者有更长的OS（97.7周*vs* 68周，*P*=0.009, 4）。此后Johnson等^[8]^报道了相似研究，一线接受VAC方案及放疗的LD-SCLC患者，随机予依托泊苷联合顺铂方案巩固治疗2个疗程（*n*=72）或观察（*n*=79），接受巩固治疗的患者同样有更长的OS（21.1个月*vs* 13.2个月，*P*=0.028）。Sculier等^[9]^开展了依托泊苷联合长春地辛作为维持治疗方案的随机对照研究，91例经一线诱导化疗方案（异环磷酰胺、依托泊苷和蒽环类药物）治疗有效的SCLC患者随机进入维持治疗组（12个疗程的依托泊苷+长春地辛）和观察组，结果显示维持化疗可明显延长患者的PFS（25周*vs* 12周，*P*=0.003），不改善OS（48周*vs* 38周，*P*=0.10）。Hanna等^[10]^于2002年公布了口服依托泊苷作为维持治疗的Ⅲ期随机对照研究结果，纳入144例一线接受依托泊苷联合异环磷酰胺及卡铂方案化疗4周期后无进展的ED-SCLC患者，随机分成两组，试验组72例，依托泊苷口服50 mg/m^2^连用21天，28天为一周期，直到进展，对照组72例不服用任何药物，结果显示依托泊苷维持治疗能明显延长患者的PFS（8.23个月vs 6.5个月，*P* < 0.001, 8），倾向于改善OS（12.2个月*vs* 11.2个月）、1年生存率（51.4% *vs* 40.3%）及3年生存率（9.1% *vs* 1.9%）。维持治疗阶段的主要不良反应为血液学毒性，非血液学毒性较轻微，大部分患者可以耐受。

ECOG 7593是一项Ⅲ期随机对照研究^[11]^，评估拓扑替康维持治疗的效果，402例初治的ED-SCLC患者一线接受4周期依托泊苷联合顺铂方案化疗，223例疾病稳定或者临床缓解的患者随机进入4周期拓扑替康（1.5 mg/m^2^/d，5天，每3周1次）维持治疗组或者观察组；拓扑替康维持治疗组对比观察组有更长的PFS（3.6个月*vs* 2.3个月，*P* < 0.001），但两组的OS无明显差异（8.9个月*vs* 9.3个月，*P*=0.43）。研究者认为：ED-SCLC患者接受拓扑替康维持治疗可以延长PFS，但不能提高OS，也不能改善生活质量；标准的4周期依托泊苷联合顺铂方案仍是一般状况良好的ED-SCLC患者一线治疗的合理选择。国内蒋玮等^[12]^报道一项拓扑替康用于SCLC维持治疗的回顾性分析，53例SCLC患者（LD-SCLC患者占58.3%），29例行拓扑替康维持或巩固治疗，24例行拓扑替康挽救治疗，两组患者的OS无明显差异（20个月*vs* 27个月，*P*=0.89）。

一项Ⅱ期临床研究^[13]^评价了经过伊立替康联合顺铂方案的诱导治疗后，伊立替康维持治疗的意义。2003年3月-2006年4月总共有120例初治的ED-SCLC患者接受伊立替康联合顺铂方案诱导化疗，45例诱导化疗完成时仍缓解的患者随机进入伊立替康维持治疗组（*n*=21）或者观察组（*n*=24），维持治疗组和观察组的中位PFS分别为12.0个月和9.9个月，OS分别17.6个月和20.5个月，两组生存数据无明显差异。研究者认为，尽管伊立替康+顺铂方案是SCLC非常有效的诱导方案，但是诱导治疗有效的患者予伊立替康继续维持治疗并不能进一步延长生存。总体而言，仅有少数维持化疗的研究^[14]^显示出生存获益，大部分维持化疗只延长了PFS，不改善OS，且有些维持化疗方案有较大的毒副反应，尤其是原方案维持。

### 2.3 化疗药物维持治疗的*meta*分析

2005年Bozcuk等^[15]^对SCLC的维持治疗进行了*meta*分析，共纳入14项有关化疗维持/巩固治疗的随机对照研究，包含了2, 550例患者，维持化疗方案不一，结果提示维持治疗可以降低患者1年（HR=0.67, 95%CI: 0.56-0.79, *P* < 0.001）及2年（HR=0.67, 95%CI: 0.53-0.86, *P* < 0.001）的死亡率，1年生存率由30%提高至39%，2年的生存率由10%提高至14%。2010年Rossi等^[16]^也对SCLC维持治疗进行了*meta*分析，纳入21项随机对照研究，共有3, 688例患者，其中，11项临床试验使用化疗药物维持，6项临床试验用干扰素（4项α-2a干扰素和2项γ-干扰素）维持，4项临床试验用其他生物制剂维持治疗。结果显示，与观察组相比，维持治疗未能改善全组患者的OS（HR=0.93, 95%CI: 0.87-1.00, *P*=0.05）和PFS（HR=0.98, 95%CI: 0.91-1.26, *P*=0.63），但可减少化疗组（HR=0.89, 95%CI: 0.81-0.98, *P*=0.02）和α-2a干扰素维持治疗组（HR =0.78, 95%CI: 0.64-0.96, *P*=0.02）患者的死亡率；维持化疗可让患者的OS延长2周，1年生存率提高了4%（由30%提高到34%）；这意味着如果让25例患者接受治疗，才能使其中1例患者的生存超过1年，OS的延长不到1个月。显而易见，虽然维持化疗略微改善了SCLC的生存时间，但这个幅度非常有限。考虑到维持化疗不可避免地会引起各种毒副反应，研究者认为对此项*meta*分析的亚组阳性结果应当谨慎看待，维持化疗不作为常规推荐，但值得继续开展维持化疗的临床研究。

## 3 免疫相关药物的维持治疗

### 3.1 干扰素

有多项随机对照研究评价了干扰素维持治疗在SCLC中价值。Mattson^[17, 18]^、Kelly^[19]^及Jett^[20]^等均发现化放疗后缓解者予α-2a干扰素维持治疗，未能明显改善OS及PFS。在前面提到的Rossi等的*meta*分析中，亚组分析结果显示，α-2a干扰素可延长患者的生存时间3.5周，1年生存率提高9%（由30%提高至39%），但因纳入病例数较少，可能存在选择偏倚，且未对LD-SCLC及ED-SCLC患者进行分别分析，其结果有待进一步验证^[16]^。

### 3.2 Ipilimumab

细胞毒性T淋巴细胞相关抗原（cytotoxic T lymphocyte associated antigen 4, CTLA-4）是T细胞活性的负调节剂。Ipilimumab是一种人源化的IgG1型抗CTLA-4单抗隆抗体，与CTLA-4结合后阻碍后者与其配体（CD80/CD86）相互作用；阻断CTLA-4信号，导致T细胞受到的负向调控信号大幅减弱，进而恢复T细胞的抗肿瘤活性。与治疗晚期黑色素瘤不同，Ipilimumab单药治疗肺癌几乎没有明显的疗效。当Ipilimumab与化疗联合时，在非小细胞肺癌（non-small cell lung cancer, NSCLC）及SCLC似乎均观察到一定程度的临床获益。Reck等^[21]^报道了Ipilimumab联合紫杉醇和卡铂方案一线及维持治疗ED-SCLC患者的随机、双盲、多中心研究结果，130例患者分为三组：Ipilimumab同步化疗治疗组、Ipilimumab阶段治疗组（先予2个疗程的诱导化疗，再同步联合化疗与Ipilimumab）及安慰剂组（单纯化疗组），前两组一线治疗无进展患者继续予Ipilimumab维持治疗，结果显示，Ipilimumab阶段治疗组的免疫相关无进展生存时间（immune-related progression-free survival, irPFS）较安慰剂组明显延长（*P*=0.03），但Ipilimumab同步治疗组的irPFS与安慰剂组无明显差异，三组的PFS和OS均无明显差异。研究者认为Ipilimumab在ED-SCLC值得开展进一步的研究。类似研究亦在NSCLC中进行，并得出类似的结果。由此，拉开了Ipilimumab治疗肺癌的Ⅲ期临床研究的序幕。CA184-156和CA184-R62研究是Ipilimumab在广泛期SCLC中开展的Ⅲ期随机对照研究，分别在全球和中国患者中进行，研究方案的设计基本类同于CA184-104研究。这些Ⅲ期随机研究的结果将会全面评估Ipilimumab在肺癌中的疗效、不良反应及治疗价值。

### 3.3 细胞免疫治疗

肿瘤的细胞免疫治疗（cellular immunotherapy, CIT）是指利用自然杀伤细胞（natural killer, NK）和细胞因子诱导的杀伤细胞（cytokine-induced killer, CIK）达到抗肿瘤的目的。一项前瞻性研究^[22]^入组58例SCLC患者研究细胞免疫治疗作为SCLC患者化疗后的维持治疗的疗效。CIT治疗组和对照组各29例患者，结果显示，两组之间的PFS并无差异，但CIT治疗组的OS明显长于对照组（20个月*vs* 11.5个月，*P*=0.005）。进一步数据分析提示，在局限期SCLC患者中，PFS在两组之间无明显差异，但CIT治疗组的OS显著长于对照组（26.5个月*vs* 11.8个月，*P*= 0.033）。而在广泛期SCLC患者中，CIT治疗组的PFS（5个月*vs* 2.7个月，*P*=0.037）和OS（14.5个月*vs* 9个月，*P*=0.038）均显著长于对照组。并且，研究提示CIT作为SCLC患者化疗后的维持治疗，耐受性良好，无明显毒副作用。本研究的结果可喜，但是CIT在SCLC维持治疗中的确切疗效还需要更大的临床数据支持。

## 4 分子靶向药物的维持治疗

近年一系列靶向药物的研究几乎覆盖了SCLC的各个分子靶点，其中绝大多数研究为小样本、非随机的Ⅰ期-Ⅱ期临床研究，其中有一些研究聚焦在维持治疗上，尤其是抗血管生成方面。

### 4.1 沙利度胺

沙利度胺具有免疫调节及抑制肿瘤血管生成的作用。2008年Lee等^[23]^报告了一项Ⅱ期临床研究，评估沙利度胺联合化疗作为一线和维持治疗药物在SCLC中的毒副反应及疗效，25例初治的SCLC患者接受沙利度胺联合依托泊苷和卡铂作为一线治疗方案，后继续使用沙利度胺维持治疗，结果患者的中位PFS为8.3个月，OS为10.1个月，沙利度胺维持治疗耐受性良好。随后在英国开展了一项双盲、安慰剂对照的Ⅲ期随机对照研究^[24]^，沙利度胺组在化疗开始时同步口服沙利度胺，化疗结束后继续口服沙利度胺作为维持治疗，沙利度胺起始剂量100 mg/d，如耐受良好加量至200 mg/d，对照组为安慰剂联合化疗及安慰剂维持治疗，总共入组724例患者，结果显示LD-SCLC患者中，安慰剂组和沙利度胺组的中位OS分别为12.1个月及13.1个月（HR=0.91, 95%CI: 0.73-1.15），沙利度胺组与安慰剂组无明显的生存差异；ED-SCLC患者结果表明，安慰剂组合沙利度胺组的中位OS分别为9.1个月和8.0个月（HR=1.36, 95%CI: 1.10-1.68），沙利度胺与化疗的联合反而缩短了患者的生存时间，增加了患者的血栓风险（肺、深静脉血栓）。此研究否定了沙利度胺在SCLC中的治疗作用。

### 4.2 贝伐珠单抗

贝伐珠单抗是作用于血管内皮生长因子的单克隆抗体。Horn等^[25]^于2009年发表了一项Ⅱ期临床研究，顺铂联合依托泊苷（cisplatin plus etoposide, EP）方案联合贝伐珠单抗一线治疗广泛期患者后继续予贝伐珠单抗维持治疗，结果患者PFS 4.7个月，OS 10.9个月。2011年Spigel等^[26]^报道了一项Ⅱ期广泛期患者的临床研究，治疗组为依托泊苷联合铂类方案化疗联合贝伐珠单抗作为一线及维持治疗药物，对照组为同样化疗方案联合安慰剂治疗，结果贝伐珠单抗组的中位PFS长于安慰剂组（5.5个月*vs* 4.4个月），但中位OS两组相似（9.4个月*vs* 10.9个月）。最近Pujol等^[27]^报道贝伐珠单抗联合化疗一线治疗广泛期SCLC的Ⅱ期-Ⅲ期研究（IFCT-0802），患者先接受2个疗程化疗，有效者再随机分组，研究组（*n*=37）贝伐珠单抗联合化疗，再予贝伐珠单抗维持治疗；对照组（*n*=37）单用化疗，两组的中位PFS分别为5.5个月和5.3个月（*P*=0.82），结论为诱导化疗后联合贝伐珠单抗未能改善广泛期SCLC的生存结果。这两项研究显示贝伐珠单抗的联合及维持治疗在广泛期SCLC的PFS改善上未能达成一致。

### 4.3 舒尼替尼

舒尼替尼是多靶点的酪氨酸激酶抑制剂。有研究^[28]^报道舒尼替尼维持治疗广泛期患者的结果，一线采用EP方案化疗，缓解者予舒尼替尼维持治疗，至疾病进展或者不可耐受，中位PFS为2.5个月，与历史文献相比，PFS并无延长。但2012年Spigel等^[29]^发表的另一项舒尼替尼的维持治疗的Ⅱ期临床研究表明对广泛期患者，一线伊立替康联合卡铂方案化疗后，疾病控制者序贯舒尼替尼维持治疗，总的1年生存率为54%，中位疾病进展时间7.6个月；研究者认为ED-SCLC有54%的1年生存率很鼓舞人心。2015年Ready等^[30]^报道一项Ⅱ期临床研究，入组ED-SCLC患者144例，一线依托泊苷联合铂类化疗后缓解者随机进入舒尼替尼维持治疗组（44例）及安慰剂组（41例）；结果显示安慰剂组及治疗组的中位PFS分别为2.1个月和3.7个月（HR=1.62, *P*=0.02），中位OS分别为6.9个月和9.0个月（HR=1.28, *P*=0.016）；舒尼替尼维持治疗组的不良反应主要：发生率 > 5%的3度不良反应有疲劳（19%）、中性粒细胞减少（14%）、白细胞下降（7%）和血小板减少（7%）；4度不良反应有胃肠道出血、胰腺炎、高钙血症及高脂血症各1例；结果表明舒尼替尼作为维持治疗用于广泛期SCLC的治疗可改善PFS，安全性好。

### 4.4 伊马替尼

伊马替尼是酪氨酸激酶抑制剂。Schneider等^[31]^在c-Kit阳性的ED-SCLC患者进行伊马替尼维持治疗的研究，一线化疗后缓解者口服伊马替尼直至病情进展或者毒性不可耐受，结果显示中位PFS为4.3个月，中位OS为7.8个月，提示伊马替尼未能使患者获益。

### 4.5 索拉非尼

索拉非尼是多靶点激酶抑制剂。2014年Sharma等^[32]^在ED-SCLC患者一线EP方案联合索拉非尼，化疗后缓解者继续索拉非尼维持治疗，维持治疗不超过12个月，中位OS为7.4个月，1年生存率为25%，结果表明索拉非尼未能延长生存时间^[29]^。

其他多靶点酪氨酸激酶抑制剂也在探索中，有一个小样本的研究评估了凡德他尼的维持治疗，但初步结果不理想；其他分子靶向药物如mTOR抑制剂CCI-779在SCLC维持治疗方面亦进行了前期探索，结果不尽人意。

纵观上述研究不难发现，现有分子靶向药物的临床试验，多数未能限定具有相应分子靶点的患者才能入组，其根本原因可能在于对SCLC的分子病理机制尚缺乏充分了解，未找到合适的治疗靶点，未能开发出真正有效的靶向药物；所以，目前SCLC分子靶向药物的维持治疗仅停留在探索研究阶段，尚无临床治疗推荐。

## 5 总结及展望

SCLC的维持治疗研究，从化疗药物、生物制剂到分子靶向药物，甚至是中医药领域^[33]^均有涉及，但结果参差不齐，临床获益有限，迄今没有一个药物或方案得到临床推荐，究其原因主要有以下两方面：首先SCLC本身是一种非常特殊的肺癌，生长速度快、短期内出现耐药，分子病理机制错综复杂，目前对此仍未充分了解。其次在SCLC治疗方面，几十年来鲜有进展，治疗药物有限；一线标准治疗方案（EP方案）已应用30余年，但至今没有新的治疗方案能超越他；拓扑替康是唯一得到批准的二线药物，具有效率低、缓解期短、毒性较大等特点；分子靶向药物至今没有突破；要寻找一个理想的维持药物或方案，符合有效、低毒的基本要求，从目前看仍有一定难度。

纵观已进行的维持治疗方面的相关研究，存在不少缺陷，如总体的研究数量有限、研究的样本量偏小、各个研究之间异质性明显等；这种各项研究之间的异质性，如疾病分期、体力状态评分、一线治疗的缓解状态、随机的方法、研究的主要目的等方面的差异将导致各项研究之间缺乏可比性，很难得出统一的结论。因此，SCLC维持治疗的进一步发展需要更深入地研究其分子病理机制，找到潜在的治疗靶点；研发高效、低毒的新药；设计严谨的随机对照研究方案；寻找真正能从维持治疗中获益的人群。
